# Development and validation of a whole-cell ELISA for serologically diagnosing *Helicobacter pylori* infection in Brazilian children and adults: a diagnostic accuracy study

**DOI:** 10.1590/1516-3180.2018.0203310818

**Published:** 2018-10-22

**Authors:** Silvio Kazuo Ogata, Margarita Camorlinga-Ponce, Celso Francisco Hernandes Granato, Maria Rachel da Silveira Rohr, Ricardo Artigiani, Elisabete Kawakami

**Affiliations:** I MD, PhD. Clinical Instructor, Discipline of Pediatric Gastroenterology, Department of Pediatrics, Escola Paulista de Medicina, Universidade Federal de São Paulo (EPM-UNIFESP), São Paulo (SP), Brazil.; II MBD. Senior Researcher, Infectious Disease Research Unit, UMAE Hospital de Pediatria, Instituto Mexicano del Seguro Social, Mexico City (DF), Mexico.; III MD, PhD. Associate Professor, Discipline of Parasitic and Infectious Diseases, Department of Medicine, Escola Paulista de Medicina, Universidade Federal de São Paulo (EPM-UNIFESP), São Paulo (SP), Brazil.; IV MD, PhD. Clinical Instructor, Discipline of Gastroenterology, Department of Medicine, Escola Paulista de Medicina, Universidade Federal de São Paulo (EPM-UNIFESP), São Paulo (SP), Brazil.; V MD, PhD. Full Professor, Discipline of Clinical Pathology, Department of Pathology, Escola Paulista de Medicina, Universidade Federal de São Paulo (EPM-UNIFESP), São Paulo (SP), Brazil.; VI MD, PhD. Full Professor, Discipline of Pediatric Gastroenterology, Department of Pediatrics, Escola Paulista de Medicina, Universidade Federal de São Paulo (EPM-UNIFESP), São Paulo (SP), Brazil.

**Keywords:** Helicobacter pylori, Serology, Enzyme-linked immunosorbent assay, Child, Adult

## Abstract

**BACKGROUND:**

Serological tests are practical, with low cost, but no noninvasive tests are available for diagnosing *Helicobacter pylori* (*H. pylori*) infection in Brazil. The aim here was to develop and validate enzyme-linked immunosorbent assay (ELISA) serological tests to detect anti-*H. pylori* immunoglobulin G antibodies, based on cultured strains from Brazilian patients.

**DESIGN AND SETTING:**

Cross-sectional, diagnostic accuracy study comparing a locally developed and validated ELISA and invasive tests among dyspeptic patients at two public hospitals in São Paulo, Brazil.

**METHODS:**

An ELISA test was prepared using whole-cell antigen from 56 strains. After genotypic characterization, it was standardized and optical density (OD) cutoffs were determined based on the serum antibody response of 100 *H. pylori*-negative samples, compared with 82 *H. pylori*-positive samples. Validation was performed on 174 symptomatic patients.

**RESULTS:**

The optimal OD cutoffs established (for monoclonal and polyclonal tests, respectively) were 0.167 and 0.164; overall ELISA sensitivity: 84.3%, 78.9%; specificity: 88.6%, 90.6%; positive predictive value (PPV): 75.4%, 80%; negative predictive value (NPV): 93.1%, 81.8%; accuracy: 87.3%, 86.2%; child and adolescent ELISA sensitivity: 74.2%, 81.8%; specificity: 90.8%, 86.7%; PPV: 66.6%, 84.3%; NPV: 95.8%, 84.8%; accuracy: 88.5%, 84.6; adult ELISA sensitivity: 84.4%, 75%; specificity: 86.9%, 93%; PPV: 81.8%, 78.3%; NPV: 88.9%, 91.8%; accuracy: 85.9%, 88.5%.

**CONCLUSION:**

The polyclonal serological test developed using local strains presented better diagnostic performance among children and adolescents, while the monoclonal test was better among adults. The results from both tests suggest that these in-house serological tests could be used to detect anti-*H. pylori* antibodies in our population, for screening purposes.

## INTRODUCTION

*Helicobacter pylori* (*H. pylori*) colonizes the stomach of more than half of the world’s population, mainly in developing countries.^1^^,^^2^ In fact, the burden of *H. pylori* infection is mostly borne by developing countries and specific high-risk groups in poor communities.^3^

This infection is associated with chronic gastritis, peptic ulcer disease and mucosa-associated lymphoid tissue (MALT) lymphoma in children and adults, and with development of gastric cancer, which occurs typically in adulthood.^4^ Although the incidence and mortality rates for gastric cancer have been slowly decreasing in many countries over the last five decades, it is still the fifth most common cause of cancer-related deaths, and more than 70% of gastric cancer cases occur in developing countries.^5^ Thus, gastric cancer will still remain an important healthcare problem over the coming decades, particularly in poor communities.

Recently, we demonstrated that *H. pylori* eradication plays a role in the treatment of chronic immune thrombocytopenic purpura (ITP) in children.^6^ Moreover, a systematic review study has shown that, among adults, detection and eradication of *H. pylori* infection should be considered in the management of patients with seemingly typical ITP.^7^

Different diagnostic methods are used to assess *H. pylori* infection. To perform the rapid urease test, culturing, polymerase chain reaction (PCR) and histological analysis, the invasive procedures of esophagogastroduodenoscopy and gastric biopsy are required. Among the methods that are usually considered “noninvasive” for *Helicobacter* infection (serological tests, C^13^-urea breath test and stool antigen test), detection of serum-specific anti-*H. pylori* antibodies is the only one available in Brazil, and this is limited to a few laboratories in big cities.

Anti-*H. pylori* immunoglobulin G (IgG) antibody detection by means of the enzyme-linked immunosorbent assay (ELISA) is the third-best method for noninvasive screening for *H. pylori* infection.^8^ It might be suggested as the first method, in high-prevalence areas, if the urea breath test and stool antigen test are not available.^9^ Furthermore, serological tests are considered to be the most efficient diagnostic method under certain clinical situations in which local changes in the stomach may affect the results from other tests (i.e. in cases of gastrointestinal bleeding, atrophic gastritis, gastric MALT lymphoma and gastric carcinoma), or when antibiotics or proton pump inhibitors are used.^8^ ELISA tests developed using *H. pylori* antigens from local samples usually present better accuracy than that of commercial tests based on non-local *H. pylori* antigen. One of the main criticisms of commercial tests is that they are widely used in any population (including in developing countries). Therefore, development of a local test has been encouraged.^10^^,^^11^^,^^12^^,^^13^^,^^14^^,^^15^^,^^16^^,^^17^ Hence, an easily performed, low-cost, locally developed and validated noninvasive test, for widespread use, might be recommendable for screening for *H. pylori* infection. In particular, use of such tests might potentially reduce the need to perform invasive tests, in a test-and-treat strategy.

## OBJECTIVE

The aims of this study were to develop and validate an ELISA-based serological test using whole-cell antigen of cultured *H. pylori* from gastric biopsies on Brazilian patients, in order to detect anti-*H. pylori* IgG antibodies in children and adults.

## METHODS

### Design and setting

This was a cross-sectional diagnostic accuracy study, in which a serological test to diagnose *Helicobacter pylori* infection was developed. The locally developed ELISA serological test was standardized and the optical density (OD) cutoff was determined based on the serum antibody responses of 100 *H. pylori*-negative uninfected patients. A cross-sectional study comparing this in-house ELISA serological test with invasive tests was conducted among 174 consecutive dyspeptic patients at two public hospitals in São Paulo, Brazil.

### Gastric biopsy samples and strain isolation

We examined a series of preserved frozen cultures (-80 °C) that had been isolated from gastric biopsy samples from 29 children and adolescents and 27 adult patients who were attended at the endoscopy units of two public hospitals: Hospital São Paulo, a university hospital, and Hospital Infantil Cândido Fontoura, a secondary-level children’s hospital, which are both in the city of São Paulo, Brazil. Antral gastric biopsies were processed and cultured in brain-heart infusion (BHI) agar (BHI Agar, BD Difco, Becton-Dickinson, NJ, USA), on plates that were incubated for 10-14 days under microaerophilic conditions, as previously described.^18^

### Virulence gene detection by means of conventional PCR

Samples of bacteria were harvested from a three-day-old culture and were suspended in 1 ml of distilled water for deoxyribonucleic acid (DNA) extraction (Wizard genomic DNA purification kit, Promega), in accordance with the manufacturer’s instructions. For *H. pylori* DNA confirmation, a PCR specific for the *H. pylori* UreA and B genes was used.^19^ PCR amplification of the VacA signal sequence and midregion, and of the gene CagA, was performed as previously described.^20^^,^^21^ Negative and positive controls were included in all reactions.

The gene CagA was identified in 27/56 (48.2%). The gene VacA s1 was observed in 41/56 (73.2%) and VacA s2 in 15/56 (26.8%). The allele VacA m1 was identified in 35/56 (60.7%) and m2 in 21/56 (39.3%): s1m1 occurred in 31/56 (55.3%), s1m2 in 10/56 (17.8%), s2m1 in 3/56 (5.3%) and s2m2 in 12/56 (21.4%).

### Antigen preparation

Whole-cell antigen from a sonicated pool (Ultrasonic Disruptor QR500W, Ultronique, Brazil) of 56 strains was obtained as previously described.^22^ Antigen preparation was adjusted to a protein concentration of 1 mg/ml,^23^ and aliquots of 100 µl were stored at -20 °C until used.

### ELISA standardization

Detection of anti-*H. pylori* IgG antibodies was, firstly, tested on 10 *H. pylori*-positive patients (five adults and five children) and 10 *H. pylori*-negative patients (five adults and five children), who formed positive and negative serum controls, respectively. Their diagnoses were based on invasive methods: the gold standard for positive diagnoses was a positive culture and/or positive histological test and a positive rapid urease test; and, for negative diagnoses, all three tests needed to be negative.

Standardization was performed as previously described by Camorlinga-Ponce et al.^23^ For each assay, the optimal antigen concentration and working serum dilution were determined based on checkerboard titrations. ELISA plates were prepared as previously described.^22^

Polyclonal and monoclonal antibodies were used to evaluate the best detection test. Polyclonal antibodies comprise collections of antibodies from different B cells that recognize multiple epitopes on the same antigen. For this reason, they provide greater sensitivity, even for detecting proteins that are present in low quantities in a sample. In contrast, monoclonal antibodies comprise antibodies from a single antibody-producing B cell. Thus, they bind with a single epitope. This is highly specific, with only a small risk of cross-reactivity, and it can provide better results in assays requiring quantification of the protein levels.

For the polyclonal test (peroxidase-conjugated polyclonal anti-human IgG antibodies; Sigma), color was developed using 100 µl of 0.4 mg/ml ortho-phenylenediamine dihydrochloride diluted in citrate-phosphate buffer and 0.03% sodium perborate (Sigma) as the substrate. The plates were incubated under dark conditions at 37 °C and, after 20 minutes, 50 µl of stop solution (2M H_2_SO_4_) was added to each well. Absorbance was read at 492 nm (Multiskan FC microplate photometer). For the monoclonal test (alkaline phosphatase-conjugated monoclonal anti-human IgG antibodies; Sigma), color was developed using 100 µl of 1 ­mg/­ml p-nitrophenylphosphate (Sigma) and absorbance was read at 405 nm (Multiskan FC microplate photometer). All reactions were performed in triplicate, and the mean of three optical density (OD) measurements was used.

Analysis on the checkerboard titration for polyclonal antibodies showed that the following were the optimal concentrations: for antigen preparation, 1:500 (2 µg/ml); for working serum dilution, 1:400; and for peroxidase-conjugated anti-human antibodies, 1:60,000. For monoclonal antibodies, the following were the optimal concentrations: for antigen preparation, 1:300 (3.3 ­µg/­ml); for working serum dilution, 1:400; and for alkaline phosphatase-conjugated anti-human antibodies, 1:25,000. The best reading time for monoclonal antibodies was 25 minutes. ELISA tests were run for four consecutive days to evaluate interoperability and reproducibility.

### Antigen specificity

The cross-reactivity of the in-house ELISA serological tests for *H. pylori* antigens was evaluated against whole-cell antigens or membrane proteins of 14 heterologous bacterial species: *Campylobacter jejuni*, *Escherichia coli*, *Pseudomonas aeruginosa* (mucoid), *Pseudomonas aeruginosa* (nonmucoid), *Burkholderia cepacia*, *Klebsiella pneumoniae, Klebsiella sp., Proteus mirabilis, Proteus vulgaris, Shigella flexneri, Shigella sp., Salmonella typhi, Salmonella enterica* and *Salmonella sp*. These reactions were performed through competitive inhibition assays, as previously described.^24^ No positive reactions were observed.

### Determination of cutoff

The optical density (OD) cutoffs for the monoclonal and polyclonal tests were determined based on the serum antibody responses of 100 samples (50 adults and 50 children and adolescents) from *H. pylori*-negative uninfected patients, based on the gold standard. Thus, a pool of these 100 serum samples was used as the negative serum control. The threshold for positivity was defined as the mean value plus three standard deviations of the optical density, as previously described.^23^

These values were compared with the values from a collection of 82 serum samples (33 adults and 49 children and adolescents) from *H. pylori*-positive infected patients, based on the gold standard, which presented OD values above the corresponding cutoff, without showing any overlapping values. A pool of these 82 samples was used as the positive serum control.

During the testing of the unknown samples, a positive serum pool was included in quadruplicate in every plate and the mean of the four OD values was used to calculate the threshold for that plate. The results from each serum sample were defined as the ratio of the OD value of the sample to the threshold value and were expressed in ELISA units (EU). Serum samples with EU > 1.0 were considered seropositive.

### Validation of in-house ELISA serological assay

Peripheral blood samples (10 ml) were collected by means of venous puncture from 174 patients on whom esophagogastroduodenoscopy had been performed. The serum was stored at -20 °C. The following were taken to be exclusion criteria: recent use of antibiotics, H2 receptor antagonists, proton pump inhibitors and/or bismuth salts, and presence of digestive or extra-digestive chronic diseases. Six gastric biopsies were taken for the rapid urease test and for histological evaluation (one from the gastric body and one from the antrum, for each of these), and for culturing (two from the antrum). The procedures were performed as previously described.^18^^,^^25^

### Statistical analysis

Qualitative variables were described in terms of their proportions, and quantitative variables were described in terms of their means and standard deviations. Analyses on continuous variables were based on the negative gold standard (negative rapid urease test, histological evaluation and culturing). The threshold for positivity was defined as the mean value plus three standard deviations of the optical density. The positive serum control was standardized using the positive gold standard (positive culture and/or positive histological evaluation and rapid urease test) and presence of a positive serological test, i.e. OD over the established cutoff value. The OD for each serum sample was determined in triplicate. The optimal OD cutoff was determined based on receiver operating characteristic (ROC) curves using different sensitivity and specificity values, and the area under the ROC curve (AUC) was estimated taking the significance level to be an accuracy value of 0.5.

The sample size calculation for in-house ELISA serological test validation was based on a prevalence of *H. pylori* infection of around 30% in children and 50% in adults in Brazil. The desirable AUC was considered to be 0.90, with a significance level of 0.05 and absolute precision of 5%. Thus, for this study, the sample size was estimated to be 78 adults and 95 children.

The sensitivity, specificity, accuracy and positive and negative predictive values of the in-house ELISA serological tests were evaluated by comparing OD values, based on cutoffs that were determined using the ROC curve, against the gold standard (rapid urease test, histological evaluation and culturing). Negative and positive likelihood ratios were evaluated.

### Ethical considerations

This study was approved by the Institutional Review Board of the Federal University of São Paulo (Universidade Federal de São Paulo), under registration number 180.606, on December 21, 2012, and under Brazil Platform registration number (CAAE) 10235612.0.0000.5505. All patients and/or their guardians were informed about the purposes of this study and signed an informed consent form.

## RESULTS

### Patients

Between November 2015 and August 2017 (20 months), 847 children and adolescents and 432 adults were evaluated by means of esophagogastroduodenoscopy at Hospital São Paulo, a university hospital, and Hospital Infantil Cândido Fontoura, a secondary-level children’s hospital, which are both in the city of São Paulo, Brazil. *H. pylori* status was defined by means of the gold standard described here, and, for validation purposes, no equivocal findings were included. To validate our in-house serological test, 174 consecutive dyspeptic patients, for whom the *H. Pylori* infection status was unknown were chosen: 96 children and adolescents (age range 2-17 years; mean: 12.2 ± 3.7 years) and 78 adult (age range 19-85 years; mean: 52.3 ± 18.1 years). The esophagogastroduodenoscopy findings were normal in 108/174 patients (62%) and abnormal in 66/174 (38%). Among the latter, 57/174 (32.7%) were *H. pylori*-positive. Among the children and adolescents of this sample, 24/96 (25%) were *H. pylori*-positive; while among the adults, 33/78 (42.3%) were *H. pylori*-positive.

### Determination of cutoff

The serum samples that were negative in the monoclonal test presented OD values ranging from 0.085 to 0.155 (mean ± standard deviation, SD: 0.11996 ± 0.017695). The threshold for positivity was 0.173. The serum samples that were negative in the polyclonal test presented OD values ranging from 0.061 to 0.148 (mean ± SD: 0.10336 ± 0.0164024). The threshold for positivity was 0.153. The serum samples (82 samples) that were positive in the monoclonal test presented OD values ranging from 0.180 (1.04 EU) to 0.464 (2.85 EU) (mean ± SD: 0.22968 (1.33 EU) ± 0.057). The serum samples (82 samples) that were positive in the polyclonal test presented OD values ranging from 0.172 (1.12 EU) to 0.437 (2.85 EU) (mean ± SD: 0.261 (1.7 EU) ± 0.06019).

Despite the threshold for positivity based on the mean for the negative results plus 3 SD in the monoclonal test (0.173) and in the polyclonal test (0.153), the optimal OD cutoff value based on ROC curve analysis was found to be 0.167 in the monoclonal test, with an area under the ROC curve of 0.9 (95% confidence interval, CI: 0.87-0.92). The optimal OD cutoff value was 0.164 in the polyclonal test, with an area under the ROC curve of 0.915 (95% CI: 0.89-0.93).

### Validation of the ELISA serological assay

Analysis on the overall seropositive results (EU > 1.0) from the monoclonal test presented little difference in values, compared with the polyclonal test, but the sensitivity and negative predictive values were slightly higher with monoclonal antibodies. When age was considered, the monoclonal test presented a better result for sensitivity among adults, but specificity was better among children and adolescents. In contrast, the polyclonal test presented a better result for sensitivity among children and adolescents. The accuracy was slightly better among children and adolescents in the monoclonal test and among adults in the polyclonal test (Table 1).

**Table 1:** Analysis on serological tests (sensitivity, specificity, accuracy, positive and negative predictive values and positive and negative likelihood ratios)

Type of test	Category	Sensitivity % (95% CI)	Specificity % (95% CI)	PPV % (95% CI)	NPV % (95% CI)	Accuracy % (95% CI)	Positive likelihood ratio	Negative likelihood ratio
Monoclonal	General	84.3 (83.4-85.3)	88.6 (85.9-87.9)	75.4 (74.5-76.3)	93.1 (92-94.1)	87.3 (86.3-88.3)	7.4	0.18
	Children and adolescents	74.2 (82.5-85.9)	90.8 (88.9-92.7)	66.6 (65.2-67.9)	95.8 (93.8-97.7)	88.5 (86.7-90)	8	0.28
	Adults	84.4 (82.3-86.5)	86.9 (84.7-89)	81.8 (79.7-83.9)	88.9 (86.7-91.1)	85.9 (83.7-88)	6.4	0.18
Polyclonal	General	78.9 (75.8-82)	90.6 (88.4-92.8)	80 (77-83)	81.8 (78.9-84.7)	86.2 (83.6-88.8)	8.4	0.23
	Children and adolescents	81.8 (77.4-86.2)	86.7 (82.8-90.5)	84.3 (80.2-88.4)	84.8 (80.7-88.8)	84.6 (80.5-88.7)	6.1	0.21
	Adults	75 (70.6-79.4)	93 (90.4-95.6)	78.3 (74.1-82.5)	91.8 (89-94.6)	88.5 (85.2-91.7)	10.7	0.27

PPV = positive predictive value; NPV = negative predictive value.

## DISCUSSION

In this study, ELISA-based serological tests to detect monoclonal and polyclonal IgG antibodies using *H. pylori* whole-cell antigen from strains isolated in our community were developed, standardized, validated and evaluated. The sensitivity and specificity of the in-house serological tests for both polyclonal antibodies (78.9% and 90.6%) and monoclonal antibodies (84.3% and 88.6%) were similar to those found by Camorlinga-Ponce et al.^23^ (85% and 89.7%). The in-house ELISA serological test that we developed did not present any cross-reactivity to heterologous bacterial species.

The sample size was appropriate, given that both the sensitivity and the specificity presented narrow 95% confidence intervals. Selectivity in relation to gastroduodenal diseases can influence the sensitivity and specificity results. A study conducted by Aziz et al.^17^ found higher OD values and this may have occurred because their study was based on peptic ulcer disease patients. In the present study, adults, children and adolescents were evaluated together, and the OD results presented little variation. However, other studies conducted on Brazilian populations have shown that age had an influence on the sensitivity and specificity of commercial tests.^26^^-^^27^

Both commercial and in-house serological tests have presented large ranges of sensitivity and specificity results. Leal et al.^14^ conducted a meta-analysis that including 42 studies in which ELISA serological tests were performed on samples from children to detect *H. pylori* infection. There were 33 studies using 19 different commercial tests and nine studies using in-house tests. They observed that the mean sensitivity was 79.2% (95% CI: 77.3-81%) and that the mean specificity was 92.4% (95% CI: 91.6-93.3%). On the other hand, an in-house serological test that was developed in Pakistan presented sensitivity of 92% and specificity of 100%, i.e. similar to or better than commercial tests.^17^

The accuracy of serological tests can also be affected by the choice of antigen that is used to prepare the in-house ELISA assay. In some studies, *H. pylori* genetic heterogeneity was taken into consideration in choosing the whole-cell lysate (with sonication).^28^^,^^29^ In our study, the strains used in the antigenic pool were verified genetically, to obtain better representativeness, including the CagA and VacA strains (alleles s1m1, s1m2, s2m1 and s2m2). The genotypic distribution in our study was similar to what has been found in other studies in Brazil.^30^^,^^31^

The diagnostic accuracy of a serological test is enhanced when the antigen pool is prepared based on multiple strains. Widmer et al.^32^ evaluated the performance of three antigenic preparations and observed that the results were superior when an enzyme immunoassay prepared with a pool of native antigens was used (sensitivity: 90-100%; specificity: 90-97%), rather than with recombinant antigen (sensitivity: 59-78%; specificity: 41-100%). Serological tests based on whole-cell antigen or whole-cell antigen lysate have also shown good performance among both children and adult patients.^11^^,^^23^^,^^24^^,^^33^

Despite the simplicity of ELISA serological tests, attention during their development is needed. In particular, the optimal antigen concentration, serum sample dilution and antibody conjugation need to be determined. These parameters should be optimized and adjusted to local conditions, to the population studied and to the method developed.^17^^,^^34^ The optimal concentrations in the present study were determined by means of the checkerboard titration method. It is very important to make these determinations because of false-positive titration results caused by high concentrations of antigens, which promote nonspecific bonds, even in negative serum. Moreover, low antigen concentrations can result in false negatives. In addition, high concentrations of conjugated antibodies may result in nonspecific reading, while low concentrations cause decreased OD. The serum concentration can also interfere with the OD results.^17^

This study was the first on our population to evaluate the serological response to anti-*H. pylori* IgG antibodies among symptomatic children, adolescents and adult patients, using an ELISA-based serological test developed using whole-cell antigen lysate from strains collected in our own community.

Ideal tests for screening purposes should present high sensitivity and good specificity. The serological test using monoclonal antibodies that was developed in this study presented sensitivity of over 80% among adults, while the serological test using polyclonal antibodies presented sensitivity of over 80% among children and adolescents. This indicates that these tests are suitable for population-based screening, especially the polyclonal test, which is characterized by greater sensitivity. On the other hand, although the higher specificity among adults in the polyclonal test, with low occurrence of false-negative results, is desirable for a diagnostic method, it is not necessary for a screening test.

## CONCLUSION

The in-house polyclonal serological test that was developed using local strains (in-house) presented better diagnostic performance than did the monoclonal test for children and adolescents. In contrast, the monoclonal test was better among adults. The results relating to accuracy, sensitivity, specificity and likelihood ratios from both tests suggest that these in-house serological tests could be used to detect anti-*H. pylori* antibodies in the Brazilian population, for screening purposes.
